# MCU-independent Ca^2+^ uptake mediates mitochondrial Ca^2+^ overload and necrotic cell death in a mouse model of Duchenne muscular dystrophy

**DOI:** 10.1038/s41598-024-57340-3

**Published:** 2024-03-21

**Authors:** Michael J. Bround, Eaman Abay, Jiuzhou Huo, Julian R. Havens, Allen J. York, Donald M. Bers, Jeffery D. Molkentin

**Affiliations:** 1 Department of Pediatrics, Cincinnati Children’s Hospital Medical Center, University of Cincinnati, 240 Albert Sabin Way, MLC 7020, Cincinnati, OH 45229-3039 USA; 2 Department of Pharmacology, University of California, Davis, CA 95616 USA

**Keywords:** Mechanisms of disease, Cardiovascular biology, Disease model

## Abstract

Mitochondrial Ca^2+^ overload can mediate mitochondria-dependent cell death, a major contributor to several human diseases. Indeed, Duchenne muscular dystrophy (MD) is driven by dysfunctional Ca^2+^ influx across the sarcolemma that causes mitochondrial Ca^2+^ overload, organelle rupture, and muscle necrosis. The mitochondrial Ca^2+^ uniporter (MCU) complex is the primary characterized mechanism for acute mitochondrial Ca^2+^ uptake. One strategy for preventing mitochondrial Ca^2+^ overload is deletion of the *Mcu* gene, the pore forming subunit of the MCU-complex. Conversely, enhanced MCU-complex Ca^2+^ uptake is achieved by deleting the inhibitory *Mcub* gene. Here we show that myofiber-specific *Mcu* deletion was not protective in a mouse model of Duchenne MD. Specifically, *Mcu* gene deletion did not reduce muscle histopathology, did not improve muscle function, and did not prevent mitochondrial Ca^2+^ overload. Moreover, myofiber specific *Mcub* gene deletion did not augment Duchenne MD muscle pathology. Interestingly, we observed MCU-independent Ca^2+^ uptake in dystrophic mitochondria that was sufficient to drive mitochondrial permeability transition pore (MPTP) activation and skeletal muscle necrosis, and this same type of activity was observed in heart, liver, and brain mitochondria. These results demonstrate that mitochondria possess an uncharacterized MCU-independent Ca^2+^ uptake mechanism that is sufficient to drive MPTP-dependent necrosis in MD in vivo.

## Introduction

Mitochondria-dependent cell death is a major contributor to several human diseases including ischemia reperfusion (I/R) injury in the heart and brain^1,2^, neurodegenerative diseases such as Alzheimer’s and multiple sclerosis^3,4^, and muscular dystrophy (MD)^5^. A key driver of mitochondria-dependent cell death is the activation of the mitochondrial permeability transition pore (MPTP)^6^. The MPTP is a high conductance channel that remains closed at homeostasis but opens in response to oxidative stress and mitochondrial Ca^2+^ overload^1^. When activated the MPTP permeabilizes the mitochondrial matrix to solutes up to 1.5 kDa, dissolves mitochondrial membrane potential, abolishes oxidative phosphorylation and ATP production, causes mitochondrial swelling and rupture, and initiates apoptosis or necrosis depending on cell type^1^. While recent work has demonstrated that the isomerase cyclophilin D (CypD) and the adenine nucleotide translocase (ANT) proteins are required for MPTP formation^5,7^, the exact molecular identity of the MPTP remains controversial^6^. As such, there is no effective therapy to directly target the MPTP and prevent mitochondria-dependent cell death in human disease.

Mitochondrial Ca^2+^ overload is known to mediate cardiomyocyte necrosis in ischemia/reperfusion (I/R) injury^8–10^. Given that the MCU-complex stands as the primary mechanism for acute mitochondrial Ca^2+^ uptake^11,12^, investigators have used mouse genetics to inhibit MCU-complex activity as a strategy to reduce cardiac I/R injury. Mice constitutively lacking the *Mcu* gene^11,12^, which show no acute mitochondrial Ca^2+^ uptake in vitro, were not protected from I/R injury in a Langendorf perfusion model^13^. In contrast, two studies performing acute cardiomyocyte-specific *Mcu* gene deletion in mice showed partial injury protection from I/R injury^14,15^. However, mice overexpressing a cardiac-specific dominant negative MCU mutant protein, which also blocked acute Ca^2+^ uptake, were not protected from acute I/R injury^16^. Conversely, cardiac overexpression of MCUb prior to I/R surgery, which encodes an endogenous MCU-complex inhibitor^17^, reduced I/R injury and adverse post-injury remodeling^18,19^. More importantly, a recent study observed that mitochondrial Ca^2+^ overload still occurs in hearts of *Mcu* deleted mice with I/R injury, albeit at a slower rate, suggesting that an alternate Ca^2+^ uptake mechanism may exist^20^. Indeed, it has also been reported that *Mcu* gene deletion does not block mitochondrial Ca^2+^ import in neonatal mouse ventricular myocytes subjected to simulated I/R conditions^21^. These results suggest that the MCU-complex may not be necessary for cell death and mitochondrial Ca^2+^ overload with cardiac I/R injury.

Duchenne and limb-girdle muscular dystrophies are muscle degenerative diseases wherein gene mutations cause sarcolemmal instability that leads to Ca^2+^ influx and myofiber necrotic cell death^22^. We have recently demonstrated that MPTP-activation is the primary driver of myofiber necrosis in MD since mice lacking both *Slc25a4* (ANT1) and *Ppif* (CypD) genes demonstrate profound MPTP desensitization and a near complete loss of MD pathology in mice^5^. We have also shown that genetic manipulations specifically causing myofiber Ca^2+^ overload in healthy muscle recapitulated necrosis and features of MD disease^23–25^. Conversely, genetic manipulations in mice that directly counteract Ca^2+^ overload reduce MD pathology^24–26^. Importantly, mitochondria from dystrophic muscle have elevated Ca^2+^ levels^5,27^. Moreover, human patients with *Micu1* mutations and *Micu1* gene deleted mice, which both show unregulated and augmented mitochondrial Ca^2+^ uptake through the MCU-complex, possess a mild MD phenotype^28,29^. Thus, unregulated Ca^2+^ influx drives muscle necrosis in MD through mitochondrial Ca^2+^ overload, which triggers MPTP opening and tissue necrosis. However, here we unexpectedly observed that the MCU-complex is not required for mitochondrial Ca^2+^ overload and skeletal muscle disease in MD, which demonstrates the physiologic existence of an MCU-independent Ca^2+^ uptake mechanism that is sufficient to cause tissue necrosis in MD even in the absence of MCU-complex function.

## Results

**Figure 1:**
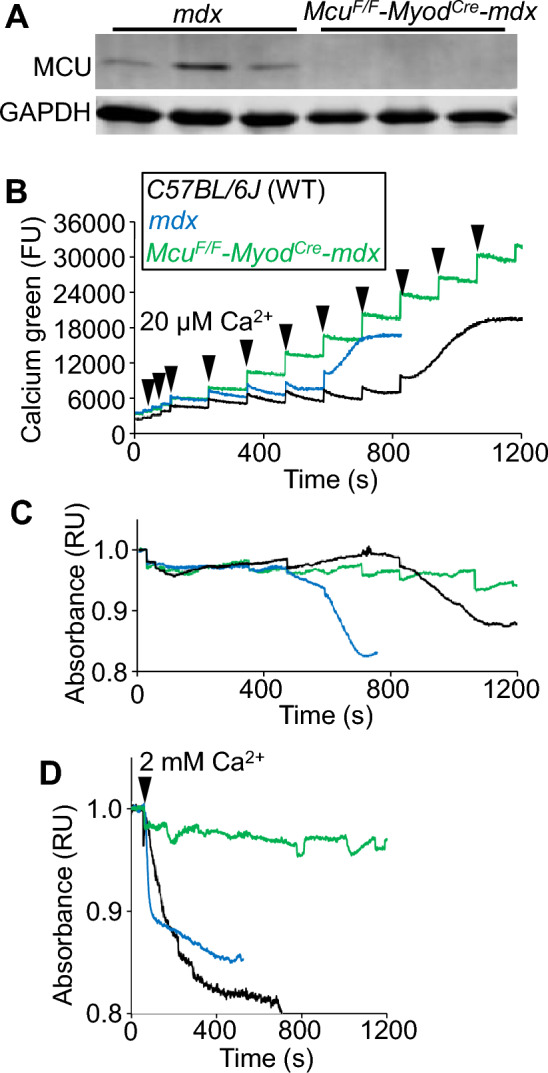
*Mcu* gene deletion protects isolated dystrophic muscle mitochondria from MPTP activation. (**A**) Western blots of MCU and GAPDH expression in *mdx* and *Mcu*^*F/F*^*-Myod*^*Cre*^*-mdx* whole protein extracts from quadriceps muscle. (**B**) Representative mitochondrial CRC assay in isolated quadriceps mitochondria from *C57BL/6 J* (WT), *mdx,* and *Mcu*^*F/F*^*-Myod*^*Cre*^*-mdx* mice. Two milligrams of mitochondria were treated with 20 µM additions of CaCl_2_ as indicated by the arrowheads. The group legend in this panel applies to the remaining 2 panels. Data are plotted as fluorescence units (FU). The presented data are representative of at least 3 independent experiments utilizing mitochondria from 3 separate mitochondrial isolations from different mice of the indicated genotypes. (**C**) Representative absorbance-based mitochondrial swelling measurements taken simultaneously with the CRC assay shown in (B) with the same Ca^2+^ additions. Data are plotted as normalized relative to absorbance (RU). **(D)** Representative absorbance-based swelling measurements of isolated quadriceps mitochondria from *mdx* and *Mcu*^*F/F*^*-Myod*^*Cre*^*-mdx* mice treated with a single 2 mM addition of CaCl_2_. Arrowhead indicates time of CaCl_2_ addition. Data are plotted as normalized relative to absorbance (RU). The presented data are representative of at least 3 independent experiments utilizing mitochondria from 3 separate mitochondrial isolations from different mice of the indicated genotypes.

Here we generated muscle-specific *Mcu* gene deleted mice on the *mdx* dystrophic background using a *Myod-Cre* knockin line and a previously characterized *Mcu*-loxP (F) targeted line (*Mcu*^*F/F*^*-Myod*^*Cre*^*-mdx*)^14,30,31^. Isolated mitochondria from skeletal muscle of *Mcu*^*F/F*^*-Myod*^*Cre*^*-mdx* mice lacked MCU protein expression (Fig. 1A) and lacked acute mitochondrial Ca^2+^ uptake in the standard in vitro Ca^2+^ retention capacity (CRC) assay (Fig. 1B). In this assay isolated mitochondria are suspended in a buffer containing an impermeant Ca^2+^ indicator dye (calcium green) and challenged with multiple Ca^2+^ boluses until either a loss of mitochondrial Ca^2+^ uptake capacity is achieved or the MPTP becomes activated which causes Ca^2+^ release and mitochondrial swelling, as observed by a change in light absorbance properties^7^. The number of Ca^2+^ additions required for MPTP activation reflects the sensitivity of the MPTP and baseline mitochondrial Ca^2+^ levels^5,7^. As previously reported^5,27^, isolated dystrophic mitochondria from muscle of *mdx* mice were sensitized to MPTP-activation relative to healthy wildtype (WT) mitochondria, as indicated by a decrease in CRC and premature mitochondrial swelling (Fig. 1B,C). In contrast, isolated mitochondria from *Mcu*^*F/F*^*-Myod*^*Cre*^*-mdx* muscle did not display MPTP activation or swelling, showing that these mitochondria cannot undergo MPTP activation without acute Ca^2+^ influx in this assay (Fig. 1C). Moreover, MPTP activation was lacking in mitochondria isolated from muscle of *Mcu*^*F/F*^*-Myod*^*Cre*^*-mdx* mice treated with 2 mM Ca^2+^, which approximates the maximum physiological Ca^2+^ concentration in vivo^32^ (Fig. 1D). These data suggest that muscle mitochondria from *Mcu*^*F/F*^*-Myod*^*Cre*^*-mdx* mice lack acute Ca^2+^ uptake and MPTP activation in vitro, which is hypothesized to protect from MD as observed in mouse models with direct MPTP inhibition by deletion of *Ppif* and/or *Slc25a4*^5,33–36^.

**Figure 2:**
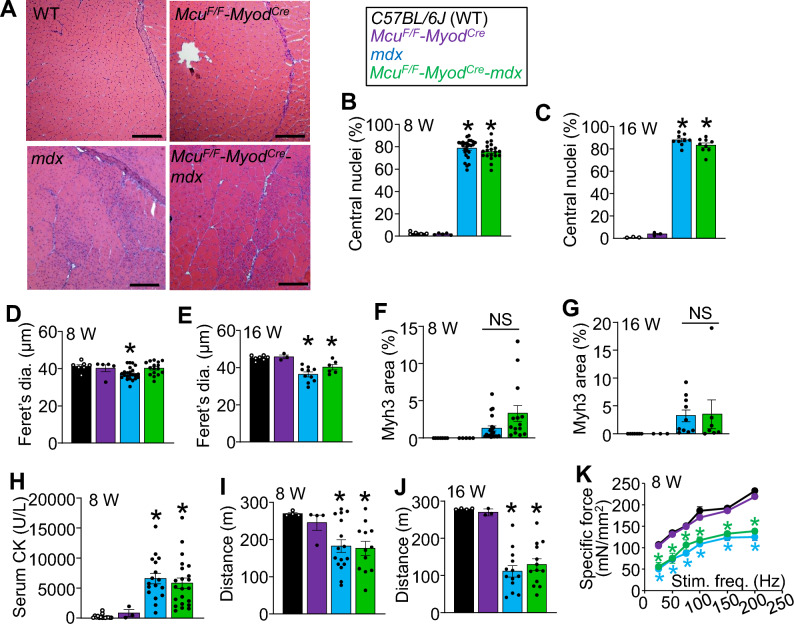
Myofiber specific *Mcu* gene deletion does not reduce muscle pathology in the *mdx* mouse model of muscular dystrophy. (**A**) Representative images of H&E-stained quadriceps histological sections from 8 wk old *C57BL/6 J* (WT), *Mcu*^*F/F*^*-Myod*^*Cre*^*, mdx*, and *Mcu*^*F/F*^*-Myod*^*Cre*^*-mdx* mice. Scale bar is 100 μm. The images are representative from at least 6 independent mice of the indicated genotypes. (**B**) Quantification of myofiber central nucleation in quadriceps histological sections from 8 wk old mice with the indicated genotypes. WT n = 5; *Mcu*^*F/F*^*-Myod*^*Cre*^ n = 5; *mdx* n = 26; *Mcu*^*F/F*^*-Myod*^*Cre*^*-mdx* n = 19 (*P ≤ 0.05 vs WT). The legend above this panel applies to all other panels. (**C**) Quantification of myofiber central nucleation in quadriceps histological sections from 16 wk old mice with the indicated genotypes. WT n = 3; *Mcu*^*F/F*^*-Myod*^*Cre*^ n = 3; *mdx* n = 9; *Mcu*^*F/F*^*-Myod*^*Cre*^*-mdx* n = 9 (*P ≤ 0.05 vs WT). (**D**) Average minimal Feret’s diameter of myofibers measured from quadriceps histological sections from 8 wk old mice of the indicated genotypes. WT n = 7; *Mcu*^*F/F*^*-Myod*^*Cre*^ n = 5; *mdx* n = 23; *Mcu*^*F/F*^*-Myod*^*Cre*^*-mdx* n = 16 (*P ≤ 0.05 vs WT). (**E**) Average minimal Feret’s diameter of myofibers measured from quadriceps sections from 16 wk old mice of the indicated genotypes. WT n = 7; *Mcu*^*F/F*^*-Myod*^*Cre*^ n = 3; *mdx* n = 10; *Mcu*^*F/F*^*-Myod*^*Cre*^*-mdx* n = 6 (*P ≤ 0.05 vs WT). (**F**) Quantification of the percent embryonic myosin heavy chain (Myh3) positive area in quadriceps histological sections taken from 8 wk old mice of the indicated genotypes. WT n = 7; *Mcu*^*F/F*^*-Myod*^*Cre*^ n = 5; *mdx* n = 21; *Mcu*^*F/F*^*-Myod*^*Cre*^*-mdx* n = 14 (NS = not significant). (**G**) Quantification of the percent Myh3 positive area in quadriceps histological sections taken from 16 wk old mice of the indicated genotypes. WT n = 7; *Mcu*^*F/F*^*-Myod*^*Cre*^ n = 3; *mdx* n = 10; *Mcu*^*F/F*^*-Myod*^*Cre*^*-mdx* n = 7 (NS = not significant). (**H**) Serum creatine kinase (CK) levels for 8 wk old mice of the indicated genotypes. WT n = 15; *Mcu*^*F/F*^*-Myod*^*Cre*^ n = 3; *mdx* n = 17; *Mcu*^*F/F*^*-Myod*^*Cre*^*-mdx* n = 22; (*P ≤ 0.05 vs WT). (**I**) Average downhill treadmill running distance for 8 wk old mice of the indicated genotypes. Maximum possible distance is 270 m. WT n = 4; *Mcu*^*F/F*^*-Myod*^*Cre*^ n = 4; *mdx* n = 16; *Mcu*^*F/F*^*-Myod*^*Cre*^*-mdx* n = 12 (*P ≤ 0.05 vs WT). (**J**) Average downhill treadmill running distance for 16 wk old mice of the indicated genotypes. Maximum possible distance is 270 m. WT n = 4; *Mcu*^*F/F*^*-Myod*^*Cre*^ n = 3; *mdx* n = 13; *Mcu*^*F/F*^*-Myod*^*Cre*^*-mdx* n = 13 (*P ≤ 0.05 vs WT). (**K**) Measurement of isometric specific force generated from the TA muscle of 8 wk old mice of the indicated genotypes. The data are graphed as active force normalized to the muscle's physiologic cross-sectional area (mN/mm^2^). WT n = 5; *Mcu*^*F/F*^*-Myod*^*Cre*^ n = 4; *mdx* n = 5; *Mcu*^*F/F*^*-Myod*^*Cre*^*-mdx* n = 3; (*P ≤ 0.05 vs WT).

Contrary to this hypothesis, *Mcu*^*F/F*^*-Myod*^*Cre*^*-mdx* mice did not show reduced MD disease features relative to *mdx* controls. Histopathology of the quadriceps was not different between *Mcu*^*F/F*^*-Myod*^*Cre*^*-mdx* and *mdx* mice (Fig. 2A). Quantitatively, muscle specific deletion of the *Mcu* gene in the *mdx* background did not reduce myofiber central nucleation, alter myofiber size distribution, or reduce myofiber regeneration as assessed by embryonic myosin heavy chain expression compared to *mdx* alone at both 8 and 16 weeks of age (Fig. 2B-G). Similarly, *Mcu*^*F/F*^*-Myod*^*Cre*^*-mdx* mice had equally elevated serum creatine kinase levels as *mdx* mice, which is a marker of whole-body muscle damage (Fig. 2H). There was also no improvement in muscle function with equal reductions in treadmill performance and muscle force achieved in *Mcu*^*F/F*^*-Myod*^*Cre*^*-mdx* and *mdx* mice compared to healthy WT animals (Fig. 2I-K). Since these same parameters are improved in dystrophic models with genetic MPTP desensitization^5,33–36^, these results demonstrate that *Mcu* gene deletion does not protect from muscle disease in the *mdx* mouse model of Duchenne MD (DMD).

**Figure 3:**
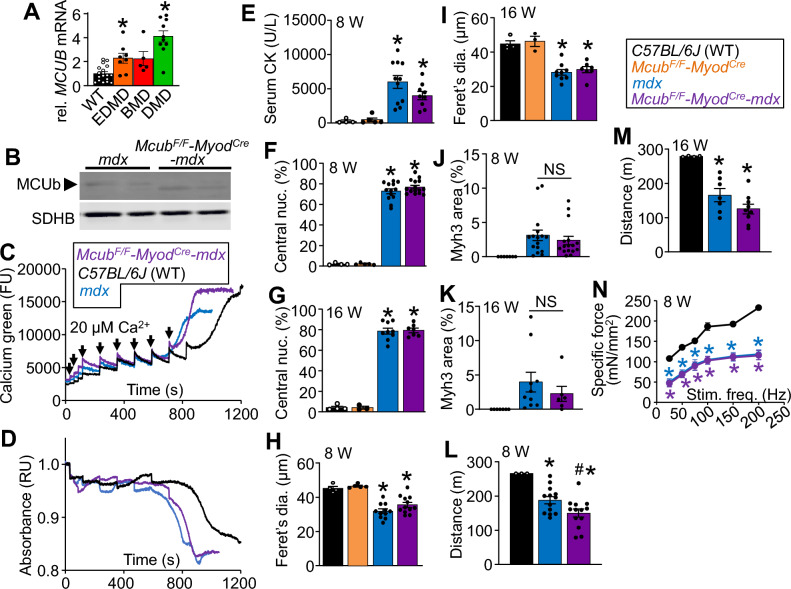
Myofiber *Mcub* gene deletion does not increase MD pathology in the *mdx* mouse model of muscular dystrophy. (**A**) *MCUB* mRNA expression in human muscle biopsies from patients with Emery-Dreifuss MD (EDMD), Becker MD (BMD), and Duchenne MD (DMD) (NCBI GEO dataset: GDS1956). *MCUB* gene expression is normalized to voltage dependent anion channel 1 (*VDAC1*) levels. WT n = 18; EMD n = 8; BMD n = 6; DMD n = 9; (*P ≤ 0.05 vs WT). (**B**) Western blots of MCUb and succinate dehydrogenase complex iron sulfur subunit B (SDHB) expression in protein extracts from isolated quadriceps mitochondria at 8 wks of age. (**C**) Representative mitochondrial CRC assay of isolated quadriceps mitochondria from *C57BL/6 J* (WT), *mdx,* and *Mcu*^*F/F*^*-Myod*^*Cre*^*-mdx* mice. Two milligrams of mitochondria were treated with 20 µM additions of CaCl_2_ as indicated by arrows. The legend above this panel applies to panel (D) as well. Data are plotted as fluorescence units (FU). The presented data are representative of at least 3 independent experiments utilizing isolated quadriceps mitochondria from 3 separate mice of the indicated genotypes. (**D**) Representative absorbance-based mitochondrial swelling measurements taken simultaneously with the CRC assay shown in (C) with the same Ca^2+^ additions. Data are plotted as normalized relative absorbance (RU). (**E**) Serum creatine kinase levels for 8 wk old mice of the indicated genotypes. WT n = 4; *Mcub*^*F/F*^*-Myod*^*Cre*^ n = 5; *mdx* n = 11; *Mcub*^*F/F*^*-Myod*^*Cre*^*-mdx* n = 9; (*P ≤ 0.05 vs WT). The legend at the top right applies to the remainder of the figure panels. (**F**) Quantification of myofiber central nucleation in quadriceps histological sections from 8 wk old mice with the indicated genotypes. WT n = 4; *Mcub*^*F/F*^*-Myod*^*Cre*^ n = 5; *mdx* n = 13; *Mcub*^*F/F*^*-Myod*^*Cre*^*-mdx* n = 15 (*P ≤ 0.05 vs WT). (**G**) Quantification of myofiber central nucleation in quadriceps histological sections from 16 wk old mice with the indicated genotypes. WT n = 4; *Mcub*^*F/F*^*-Myod*^*Cre*^ n = 3; *mdx* n = 9; *Mcub*^*F/F*^*-Myod*^*Cre*^*-mdx* n = 7 (*P ≤ 0.05 vs WT). (**H**) Average minimal Feret’s diameter of myofibers measured from quadriceps sections from 8 wk old mice of the indicated genotypes. WT n = 4; *Mcub*^*F/F*^*-Myod*^*Cre*^ n = 5; *mdx* n = 11; *Mcub*^*F/F*^*-Myod*^*Cre*^*-mdx* n = 12 (*P ≤ 0.05 vs WT). (**I**) Average minimal Feret’s diameter of myofibers measured from quadriceps sections from 16 wk old mice of the indicated genotypes. WT n = 4; *Mcub*^*F/F*^*-Myod*^*Cre*^ n = 3; *mdx* n = 9; *Mcub*^*F/F*^*-Myod*^*Cre*^*-mdx* n = 7 (*P ≤ 0.05 vs WT). (**J**) Quantification of the percent Myh3 positive area in quadriceps histological sections taken from 8 wk old mice of the indicated genotypes. WT n = 7; *mdx* n = 16; *Mcub*^*F/F*^*-Myod*^*Cre*^*-mdx* n = 16 (*P ≤ 0.05 vs WT) (**K**) Quantification of the percent Myh3 positive area in quadriceps histological sections taken from 16 wk old mice of the indicated genotypes. WT n = 7; *mdx* n = 10; *Mcub*^*F/F*^*-Myod*^*Cre*^*-mdx* n = 6 (*P ≤ 0.05 vs WT) (**L**) Average downhill treadmill running distance in meters (m) for 8 wk old mice of the indicated genotypes. Maximum possible distance is 270 m. WT n = 4; *mdx* n = 13; *Mcub*^*F/F*^*-Myod*^*Cre*^*-mdx* n = 13 (*P ≤ 0.05 vs WT; #*P* ≤ 0.05 vs WT and *mdx*) (**M**) Average downhill treadmill running distance for 16 wk old mice of the indicated genotypes. Maximum possible distance is 270 m. WT n = 4; *mdx* n = 7; *Mcub*^*F/F*^*-Myod*^*Cre*^*-mdx* n = 9 (*P ≤ 0.05 vs WT) (**N**) Measurement of isometric specific force generated from the TA muscle of 8 wk old mice with the indicated genotypes. The data are graphed as active force (mN) normalized to the muscle's physiologic cross-sectional area in mm^2^. WT n = 5; *mdx* n = 4; *Mcub*^*F/F*^*-Myod*^*Cre*^*-mdx* n = 5; (*P ≤ 0.05 vs WT).

MCUb is a dominant negative regulator of the MCU-complex that can be induced by stress such as ischemia^18^, fasting^37^, and metabolic dysfunction^38^. It has been previously reported that *Mcub* gene and protein expression is induced in muscle from *mdx* mice and that MCU-complex function is altered in MD^27^. To confirm the relevancy of MCUb to MD we examined a publicly available human muscle disease biopsy dataset (NCBI GEO dataset: GDS1956) for changes in mRNA levels^39^ and observed that *MCUB* gene expression is significantly increased in human MD disease, including DMD (Fig. 3A). This suggests that MCUb induction could represent a physiologically relevant mechanism to limit MCU-complex Ca^2+^ uptake and reduce mitochondrial Ca^2+^ overload, possibly playing a protective role in MD. We therefore generated muscle-specific *Mcub* gene deleted mice in the *mdx* dystrophic background (*Mcub*^*F/F*^*-Myod*^*Cre*^*-mdx*)^30,37^, which showed a loss of MCUb protein in adult muscle (Fig. 3B). However, muscle specific loss of *Mcub* did not alter CRC activity or mitochondrial swelling versus *mdx* control mitochondria (Fig. 3C,D). Muscle-specific deletion of the *Mcub* gene also failed to alter elevated serum CK levels, change muscle histopathology, change myofiber central nucleation, alter myofiber size, nor was embryonic myosin heavy chain positive areas altered versus *mdx* mice at 8 and 16 weeks of age (Fig. 3E-K). However, 8-week-old *Mcub*^*F/F*^*-Myod*^*Cre*^*-mdx* mice did show a slight but significant reduction in treadmill performance, which did not persist to 16 weeks of age (Fig. 3L,M). There was also no difference in direct muscle force production between the tibialis anterior (TA) muscle of *Mcub*^*F/F*^*-Myod*^*Cre*^*-mdx* and *mdx* mice at 8 weeks of age (Fig. 3N). We have previously shown that *Mcub* gene deletion does not cause muscle pathology in healthy mice^37^. Collectively, these results indicate that loss of *Mcub* does not alter DMD-like disease in the *mdx* mouse, again suggesting that acute Ca^2+^ influx by the MCU-complex is not necessary for muscle disease with MD.

**Figure 4:**
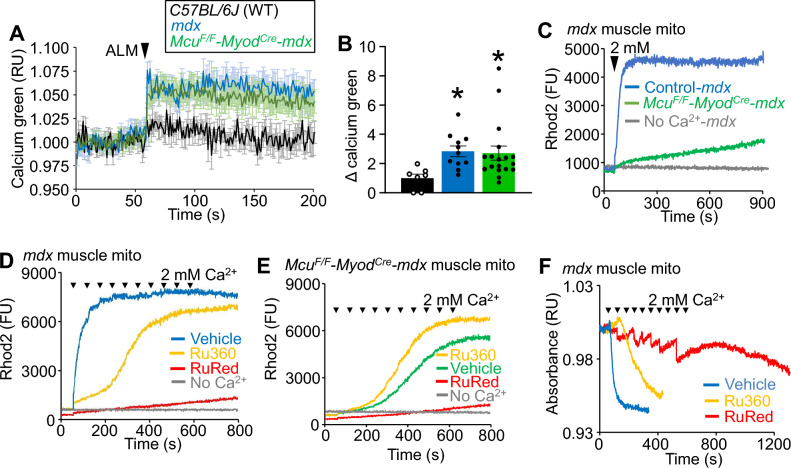
Isolated dystrophic muscle mitochondria exhibit MCU-independent Ca^2+^ uptake. (**A**) Mitochondrial Ca^2+^ assay of isolated mitochondria from WT, *mdx,* and *Mcu*^*F/F*^*-Myod*^*Cre*^*-mdx* quadriceps. Two milligrams of mitochondria were treated with 40 μM alamethicin (ALM, arrowhead) to release mitochondrial Ca^2+^ content. Data are plotted as average normalized relative calcium green buffer relative fluorescence units (RU) ± SEM. WT n = 8; *mdx* n = 11; *Mcu*^*F/F*^*-Myod*^*Cre*^*-mdx* n = 18. (**B**) Quantification of the peak Ca^2+^ released following ALM permeabilization in (A). Data are the change (Δ) from baseline to peak normalized calcium green fluorescence. WT n = 8; *mdx* n = 11; *Mcu*^*F/F*^*-Myod*^*Cre*^*-mdx* n = 18; (*P ≤ 0.05 vs WT). (**C**) Representative mitochondrial Ca^2+^ uptake trace following a single 2 mM addition of CaCl_2_ in isolated mitochondria from quadriceps of *mdx* and *Mcu*^*F/F*^*-Myod*^*Cre*^*-mdx* mice at 8 wks of age. Arrowhead indicates time of CaCl_2_ addition. Rhod2 is a mitochondria-targeted Ca^2+^ sensor dye that measures internal mitochondrial matrix Ca^2+^ levels. Data are plotted as fluorescence units (FU). The presented data are representative of at least 3 independent experiments utilizing mitochondria from 3 separate isolations from 3 mice of the indicated genotypes. This is true for all representative data presented throughout this figure. (**D**) Representative mitochondrial Ca^2+^ uptake from isolated *mdx* quadriceps mitochondria treated with 10 pulses of 2 mM CaCl_2_ (arrows). The Rhod2 indicator shows Ca^2+^ levels within mitochondria. Mitochondria were treated with 5 μM Ru360 or 1 µM ruthenium red (RuRed), or no CaCl_2_ as indicated by the figure legend. Data are plotted as fluorescence units (FU). (**E**) Representative mitochondrial Ca^2+^ uptake measured with Rhod2 in quadriceps mitochondria isolated from *Mcu*^*F/F*^*-Myod*^*Cre*^*-mdx* mice. The mitochondria were treated with 10 pulses of 2 mM CaCl_2_ (arrowheads). *Mcu*^*F/F*^*-Myod*^*CRE*^*-mdx* mitochondria were treated with 5 μM Ru360, 1 µM RuRed, or no CaCl_2_. Data are plotted as fluorescence units (FU). (**F**) Representative absorbance-based mitochondrial swelling measurements in skeletal muscle mitochondria isolated from *mdx* mice treated with 10 pulses of 2 mM CaCl_2_ until completion of the protocol or mitochondrial swelling was observed. Arrowheads denote the time of each 2 mM CaCl_2_ addition. *mdx* mitochondria were treated with 5 μM Ru360 or 1 µM RuRed. Data are plotted as normalized relative absorbance units (RU).

The finding that MCU functional loss does not alter MD pathology despite an apparent loss of acute Ca^2+^ uptake and MPTP activation in isolated mitochondria represents an interesting puzzle: how does MPTP-dependent necrosis persist without acute MCU-complex dependent mitochondrial Ca^2+^ uptake? One possibility is that *Mcu* gene deletion does not actually prevent mitochondrial Ca^2+^ overload that occurs during MD^5,27^. Indeed, direct measurement of mitochondrial Ca^2+^ levels isolated from muscle of *Mcu*^*F/F*^*-MyoD*^*CRE*^*-mdx* mice showed significantly elevated Ca^2+^ levels compared to WT mice, that was similar to the elevation observed in *mdx* control mice (Fig. 4A,B). This confirms that the MCU-complex is not required for mitochondrial Ca^2+^ overload in muscle from *mdx* mice and suggests the existence of additional MCU-independent Ca^2+^ uptake mechanisms that are sufficient to drive pathological Ca^2+^ uptake in MD.

Given that mitochondrial Ca^2+^ overload persists in skeletal muscle from *Mcu*^*F/F*^*-MyoD*^*CRE*^*-mdx* mice, and that MD is a disease of chronic Ca^2+^ overload^40^, we designed experiments to detect the presence of MCU-independent Ca^2+^ uptake in dystrophic muscle mitochondria. For these experiments a mitochondria-targeted, cell permeant Ca^2+^ indicator dye (Rhod2-AM)^41^ was used to measure mitochondrial matrix Ca^2+^. In this assay, Ca^2+^ uptake is measured by an increase in mitochondrial fluorescence. By utilizing this approach, slow mitochondrial Ca^2+^ uptake under high (> 1 mM) Ca^2+^ conditions can be measured, which would normally saturate the Ca^2+^ indicator used in the standard CRC assay. Using this new approach, mitochondria from skeletal muscle of *Mcu*^*F/F*^*-Myod*^*Cre*^*-mdx mice* exhibited a slow, but measurable Ca^2+^ uptake with 2 mM Ca^2+^ stimulation, which approximates maximal in vivo Ca^2+^ concentration^32^ (Fig. 4C).

To better characterize this MCU-independent slow Ca^2+^ uptake observed in isolated muscle mitochondria from *mdx* mice, we again modified this assay to now employ multiple 2 mM Ca^2+^ pulses. Under these elevated acute conditions substantial Ca^2+^ uptake in isolated muscle mitochondria from *Mcu*^*F/F*^*-Myod*^*Cre*^*-mdx* mice was observed, which had distinct kinetics from the Ca^2+^ uptake observed in *mdx* mitochondria containing the MCU-complex (Fig. 4D,E). When isolated muscle mitochondria from *mdx* mice were treated with Ru360, a specific small molecule inhibitor of MCU-complex Ca^2+^ uptake^42^ (Supplemental Fig. S1A), slow Ca^2+^ uptake dynamics similar to muscle mitochondria from *Mcu*^*F/F*^*-Myod*^*Cre*^*-mdx* mice was observed (Fig. 4D,E). This result suggests that whatever mediates this slow Ca^2+^ uptake process that occurs in the absence of MCU is not due to genetic compensation, given that acute Ru360 treatment of isolated mitochondria reveals the same slow Ca^2+^ influx profile as *Mcu* gene deletion. The broad-acting nonspecific Ca^2+^ transport inhibitor ruthenium red (RuRed) was also used, a drug capable of blocking multiple Ca^2+^ transporters including the MCU-complex^43–48^ (Supplemental Fig. S1A). RuRed treatment inhibited total mitochondrial Ca^2+^ uptake and swelling better than Ru360, including inhibition of the slow Ca^2+^ uptake process in muscle mitochondria from *Mcu*^*F/F*^*-Myod*^*Cre*^*-mdx* mice (Fig. 4D-F). RuRed can possess some degree of mitochondrial toxicity that may cause indirect inhibition of Ca^2+^ uptake^49^. We therefore treated isolated muscle mitochondria from *Mcu*^*F/F*^*-Myod*^*Cre*^*-mdx* mice with RuRed and then added ETH-129, a Ca^2+^ specific chemical ionophore that restores Ca^2+^ uptake in mitochondria lacking MCU^50^, which importantly is not inhibited by RuRed^51^. ETH-129 restored rapid mitochondrial Ca^2+^ uptake in RuRed-inhibited *Mcu*^*F/F*^*-Myod*^*Cre*^*-mdx* mitochondria to uninhibited levels showing that RuRed treated mitochondria are still capable of Ca^2+^ uptake (Supplemental Fig. S1B). These results demonstrate that skeletal muscle mitochondria possess a slow, MCU-independent Ca^2+^ uptake mechanism that functions in vivo at baseline and during dystrophic disease.

**Figure 5:**
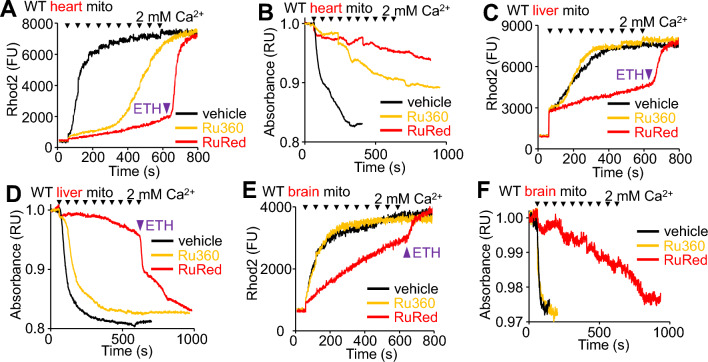
Isolated wildtype heart, liver, and brain mitochondria exhibit MCU-independent Ca^2+^ uptake. (**A**) Representative mitochondrial Ca^2+^ uptake in isolated heart mitochondria from WT mice treated with 10 pulses of 2 mM CaCl_2_ (arrowheads). Mitochondria were treated with 5 μM Ru360 or 1 µM RuRed followed by 10 μM ETH-129. Purple arrowhead indicates time of ETH-129 addition. Data are plotted as fluorescence units (FU). The presented data are representative of at least 3 independent experiments utilizing mitochondria from 3 separate isolations from different mice of the indicated genotypes. This is true for all representative data presented throughout this figure. (**B**) Representative absorbance-based mitochondrial swelling measurements of isolated heart mitochondria from WT mice treated with 10 pulses of 2 mM CaCl_2_ (arrowheads) until completion of the protocol or mitochondrial swelling was observed. Mitochondria were treated with 5 μM Ru360 or 1 µM RuRed. Data are plotted as normalized relative absorbance (RU). (**C**) Representative mitochondrial Ca^2+^ uptake in isolated liver mitochondria from WT mice treated with 10 pulses of 2 mM CaCl_2_ (arrowheads). Mitochondria were treated with 5 μM Ru360 or 1 µM RuRed followed by 10 μM ETH-129. Purple arrowhead indicates time of ETH-129 addition. Data are plotted as fluorescence units (FU). (**D**) Representative absorbance-based mitochondrial swelling measurements of isolated liver mitochondria from WT mice treated with 10 pulses of 2 mM CaCl_2_ (arrowheads) until completion of the protocol or mitochondrial swelling was observed. Mitochondria were treated with 5 μM Ru360 or 1 µM RuRed as indicated by the figure legend. RuRed treated liver mitochondria were treated with 10 μM ETH-129 as indicated by the purple arrowhead. Data are plotted as normalized relative absorbance (RU). (**E**) Representative mitochondrial Ca^2+^ uptake in isolated brain mitochondria from WT mice treated with 10 pulses of 2 mM CaCl_2_ (arrowheads). Mitochondria were treated with 5 μM Ru360 or 1 µM RuRed followed by 10 μM ETH-129 as indicated with the purple arrowhead. Data are plotted as fluorescence units (FU). (**F**) Representative absorbance-based mitochondrial swelling measurements of isolated brain mitochondria from WT mice treated with 10 pulses of 2 mM CaCl_2_ (arrowheads) until completion of the protocol or mitochondrial swelling was observed. Mitochondria were treated with 5 μM Ru360 or 1 µM RuRed as indicated by the figure legend. Data are plotted as normalized relative absorbance (RU).

Since cardiomyocytes lacking the *Mcu* gene still show increased mitochondrial matrix Ca^2+^ during ischemia^20^, here Ca^2+^ uptake within isolated cardiac mitochondria was also examined for this slow MCU-independent Ca^2+^ influx. Cardiac mitochondria treated with Ru360 did not exhibit acute Ca^2+^ uptake in vitro in a standard CRC assay (40 μM Ca^2+^ additions) (Supplemental Fig. S1C) but did possess the same slow MCU-independent Ca^2+^ uptake during the high Ca^2+^ stimulation protocol, which again was blocked with RuRed (Fig. 5A and Supplemental Fig. S1C). Cardiac mitochondria treated with Ru360 exhibited mitochondrial swelling with high Ca^2+^, but RuRed prevented this effect (Fig. 5B). In isolated liver mitochondria Ru360 also blocked acute Ca^2+^ uptake in a standard CRC assay (Supplemental Fig. S1D), however it did not overtly inhibit Ca^2+^ uptake during the high Ca^2+^ stimulation protocol (Fig. 5C). Conversely, RuRed treatment inhibited Ca^2+^ uptake in both standard CRC and high Ca^2+^ uptake conditions (Fig. 5C and Supplemental Fig. S1D). RuRed but not Ru360 was also able to prevent swelling in liver mitochondria (Fig. 5D). Similar to liver mitochondria, Ru360 treatment blocked Ca^2+^ uptake in brain mitochondria during a standard CRC (Supplemental Fig. S1E) but did not reduce Ca^2+^ uptake during the high Ca^2+^ stimulation protocol (Fig. 5E). RuRed treatment blocked Ca^2+^ uptake under standard CRC conditions in brain mitochondria (Supplemental Fig. S1E) and reduced Ca^2+^ uptake during the high Ca^2+^ protocol (Fig. 5E). However, substantial Ca^2+^ uptake was still observed in isolated brain mitochondria treated with RuRed (Fig. 5E). Similarly, Ru360 treatment did not block swelling in brain mitochondria and RuRed treatment delayed but did not prevent swelling in brain mitochondria (Fig. 5F). These experiments demonstrate that heart, liver, and brain mitochondria also possess MCU-independent Ca^2+^ uptake and suggest that there may be tissue specific differences in this process.

**Figure 6:**
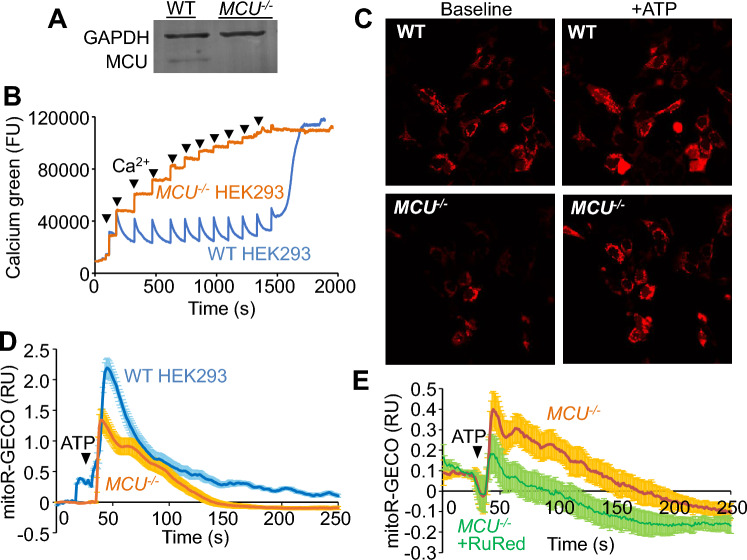
MCU-independent Ca^2+^ uptake observed in vivo in intact cells. (**A**) Western blots of MCU and GAPDH expression in WT HEK293T cells (WT) and *MCU*^*-/-*^ HEK293T cells. (**B**) Representative CRC assay of isolated mitochondria from WT and *MCU*^*-/-*^ HEK293T cells. Two milligrams of mitochondria were treated with 20 µM additions of CaCl_2_ (arrowheads). Data are plotted as fluorescence units (FU). The presented data are representative of at least 3 independent experiments utilizing mitochondria from 3 separate isolations. (**C**) Representative images of WT and *MCU*^*-/-*^ HEK293T cells transfected with a plasmid encoding mitoR-GECO, which generates red fluorescence at baseline (Baseline) due to homeostatic Ca^2+^, while peak mitochondrial Ca^2+^ fluorescence can be seen with 250 μM ATP (+ ATP) treatment. The presented images are representative of at least 5 independent experiments. Four-hundred times magnification is shown for each image. (**D**) Representative trace of normalized average mitoR-GECO mitochondrial fluorescence for WT and *MCU*^*-/-*^ HEK293T cells following treatment with 250 μM ATP (as in C). Arrowhead indicates time of ATP addition. WT n = 20 cells; *MCU*^*-/-*^ n = 20 cells. Data are plotted as normalized relative fluorescence units (RU) ± SEM. The data trace is representative of at least 5 independent experiments. (**E**) Normalized average mitoR-GECO fluorescence for *MCU*^*-/-*^ HEK293T cells treated with vehicle or 25 μM RuRed following addition of 250 μM ATP (arrowhead). WT n = 4 experiments (12–24 cells/experiment); *MCU*^*-/-*^ n = 3 experiments (20–30 cells/experiment). Data are plotted as normalized relative fluorescence units (RU) ± SEM.

A HEK293T human kidney-based cell line lacking the *MCU* gene was generated using CRISPR-Cas9 (*MCU*^*-/-*^ cells), which showed a loss of MCU protein (Fig. 6A). Isolated mitochondria from *MCU*^*-/-*^ cells lacked acute Ca^2+^ uptake within the range of the standard CRC assay (Fig. 6B). Mitochondrial Ca^2+^ measurements were also examined in cells using mitoR-GECO, a mitochondria-targeted red fluorescent Ca^2+^ sensor protein previously used in intact systems^20^. Control and *MCU*^*-/-*^ cells were stimulated with exogenous ATP to induce Ca^2+^ release in the cytoplasm, which generated a clear increase in mitochondrial mitoR-GECO fluorescence in both (Fig. 6C), indicating an equal presence of mitochondrial Ca^2+^ uptake in both cell types. *MCU*^*-/-*^ cells possessed Ca^2+^ uptake, however, the relative amplitude of the Ca^2+^ transient was smaller than in WT cells (Fig. 6D). *MCU*^*-/-*^ cells were then treated with RuRed, which reduced the MCU-independent Ca^2+^ uptake mechanism observed in intact cells lacking MCU protein (Fig. 6E). Collectively these results demonstrate that MCU-independent Ca^2+^ uptake occurs within intact cells, perhaps with greater efficiency than in isolated mitochondria. These results further suggest that MCU-independent Ca^2+^ uptake likely contributes to physiological and pathological regulation of mitochondrial Ca^2+^.

## Discussion

Neither *Mcu* nor *Mcub* muscle specific deletion altered MD pathology in the *mdx* mouse, suggesting that the MCU-complex is not required for Ca^2+^ overload induced disease at the level of the mitochondria in vivo. Moreover, *Mcu* gene deletion did not prevent or decrease mitochondrial Ca^2+^ overload that is known to occur in dystrophic muscle. These results and others from I/R injury experiments in the heart suggest that mitochondria have an MCU-independent Ca^2+^ uptake mechanism that is sufficient to mediate MPTP activation and necrosis in vivo. Indeed, a slow MCU-independent Ca^2+^ uptake mechanism was observed in isolated mitochondria from muscle that becomes significant in response to higher Ca^2+^ concentrations. This MCU-independent Ca^2+^ uptake was also detected in isolated mitochondria from heart, liver, brain, and cultured HEK293T cells suggesting that it is a general feature of mitochondria that is not limited to skeletal muscle or disease states. Collectively these data provide evidence of an unidentified MCU-independent Ca^2+^ uptake mechanism that appears to also function in necrotic disease states associated with mitochondrial Ca^2+^ overload.

The presence of an alternate mitochondrial Ca^2+^ uptake (AMCU) mechanism has previously been proposed by multiple groups^14,15,52–54^ and would explain several discrepancies in the mitochondrial Ca^2+^ homeostasis literature. Mice constitutively lacking the *Mcu* gene are viable^13,55^ in an outbred strain background, yet it should not be possible to have viable mitochondria without Ca^2+^. Moreover, many *Mcu* gene deletion models record no change^14,15^ or relatively minor decreases^13^ in baseline mitochondrial matrix Ca^2+^. The presence of a slow homeostatic Ca^2+^ uptake mechanism could explain how mitochondria lacking *Mcu* maintain baseline matrix Ca^2+^ levels and how *Mcu*^*-/-*^ mice survive in the first place. Moreover, multiple studies reported that *Mcu* gene deletion did not prevent mitochondria-dependent cell death nor substantially alter long-term mitochondrial Ca^2+^ homeostasis in the heart^13–16^, the brain^56,57^, and brown adipose tissue^58^. Moreover, cardiac mitochondria lacking *Mcu* exhibit reduced but substantial Ca^2+^ uptake during I/R experiments^20,21^. The fact that MCU-independent Ca^2+^ uptake is sufficient to drive mitochondria-dependent cell death demonstrates that this AMCU mechanism is physiologically significant and that the current model of mitochondrial Ca^2+^ homeostasis lacks a major and fundamental component.

However, the finding that MCU-independent Ca^2+^ uptake is sufficient for mitochondrial overload and cell death in MD does not exclude a role for the MCU-complex in necrosis. Since the MCU-complex provides rapid Ca^2+^ uptake under normal conditions, it is likely that a significant portion of mitochondrial Ca^2+^ overload is normally conveyed by the MCU-complex. Indeed, *Micu1* gene mutation or deletion, which causes unregulated MCU-complex Ca^2+^ uptake, causes mild MD in otherwise healthy humans and mice^28,29^, which supports a role for the MCU-complex in cell death. However, our results show that Ca^2+^ overload and MD pathology are not altered in the absence of MCU, which demonstrates that the MCU-complex is not required for this process in MD. MPTP-dependent cell death is regulated by total matrix Ca^2+^ regardless of the molecular identity of the Ca^2+^ transporter, therefore we hypothesize that multiple mitochondrial Ca^2+^ transporters or exchangers work in concert to regulate cell death in vivo. Future experiments will be required to quantify the relative contributions of MCU-dependent and MCU-independent Ca^2+^ uptake in select disease contexts.

One important caveat of the current study is the known disconnect between acute in vitro measurement of MPTP-sensitivity in isolated mitochondria and actual in vivo MPTP dynamics in cells or tissues. The standard in vitro CRC assay and absorbance-based swelling assay are tailored for measuring acute MCU-dependent Ca^2+^ influx and MPTP opening given their rapid kinetics. In the absence of MCU these assays provide a null result that resembles MPTP desensitization but does not translate into physiological protection from necrosis. These assays do not account for chronic Ca^2+^ overload that occurs in MD^40^ or gradual mitochondrial Ca^2+^ accumulation during cardiac I/R injury^1,8,20^. Indeed, as discussed above, genetic deletion of known components that directly affect or constitute the MPTP can be remarkably protective in preventing tissue necrosis in mouse models of MD or cardiac I/R injury^5,33–35,59–62^. As such, these past results with MPTP targeted mice support the observation that acute MCU-dependent Ca^2+^ influx is not necessary for MPTP in vivo, further validating the existence of another mitochondrial Ca^2+^ influx pathway.

The focus of this study was to understand the role of mitochondrial Ca^2+^ uptake in MD disease. However, it is known that dystrophic myofibers have both chronic Ca^2+^ overload^40^ and elevated oxidative stress^63^, which can both activate the MPTP^1^. Several studies have demonstrated that reducing oxidative stress can decrease MD pathology in animal models^64–68^, despite the fact that antioxidant therapies have yet to provide a clinically meaningful benefit in human DMD patients^69^. A recent study has shown that elevated mitochondrial Ca^2+^ is required for MPTP activation, and that oxidative stress plays a regulatory role wherein it lowers the Ca^2+^ threshold required for MPTP formation^1,21^. It has also been proposed that while Ca^2+^ overload occurs during cardiac I/R injury, the burst of reactive oxygen species during reperfusion may be a critical trigger of MPTP activation in the heart^21^. Thus, we hypothesize that oxidative stress in MD could sensitize MPTP opening such that slow MCU-independent Ca^2+^ uptake can now more efficiently activate the MPTP.

While this and other studies have demonstrated that necrotic cell death can occur independently of MCU, there is strong evidence that the MCU-complex has non-redundant roles in energy metabolism. In this functional model, it is known that transient or acute Ca^2+^ fluxing within the mitochondria can directly reprogram oxidative phosphorylation. Cardiac-specific *Mcu* gene deletion reduced acute functional and metabolic responses to beta-adrenergic signaling^13–16^. Similarly, both acute and long-term *Emre* cardiac gene deletion, which disrupts mammalian MCU-complex function^70,71^, resulted in impaired beta-adrenergic metabolic stimulation^72^. The pyruvate dehydrogenase complex is a primary metabolic target of mitochondrial matrix Ca^2+^, which is the key regulator for pyruvate (and by extension glucose) utilization in the tricarboxylic acid cycle^73^. Both muscle- and heart-specific *Mcu* gene deletion results in a tissue-specific shift away from oxidative glucose metabolism with a concomitant increase in fatty acid oxidation rates^74–76^. Conversely, muscle specific *Mcub* gene deletion results in increased glucose oxidation rates and reduced fatty acid utilization in skeletal muscle^37^. Hence, the MCU-complex seems ideally suited as an acute metabolic Ca^2+^ control point, but perhaps is not required for setting the baseline homeostatic Ca^2+^ that can influence MPTP and tissue necrosis.

While this study implicates an MCU-independent Ca^2+^ uptake mechanism in contributing to tissue necrosis in skeletal muscle with MD disease, the identity of the proteins or channels involved in this process are unclear. The results suggest that this activity is likely caused by a Ca^2+^ transporter given the effects of Ru360 versus RuRed, the latter of which can reduce MCU-independent Ca^2+^ uptake. As to the potential identity of this AMCU effector, one hypothesis is that it may represent a “reverse” mode activity of the mitochondrial Na^+^/Ca^2+^/Li^+^ exchanger (NCLX), which has been implicated in cardiac mitochondrial Ca^2+^ uptake during ischemia^21^. This concept may be more complicated as it is also believed that NCLX is electrogenic^77,78^, meaning that it is unlikely to drive Ca^2+^ uptake in charged mitochondria. Recently the protein TMEM65 has been implicated in Na^+^/Ca^2+^ exchange^79–81^, although it is currently unclear if it regulates NCLX or drives Na^+^/Ca^2+^ exchange directly. Future studies are required to determine if TMEM65 is capable of “reverse” mode activity, and whether it is involved in MCU-independent Ca^2+^ uptake. Another candidate is the mitochondrial H^+^/Ca^2+^ exchanger, since reductionist studies have demonstrated it can cause Ca^2+^ influx or efflux depending on assay conditions^82,83^. The exact identity underlying mitochondrial H^+^/Ca^2+^ remains unknown, however genetic deletion of the two leading candidates *Tmbim5*^84^ and *Letm1*^83,85^ both exhibit reduced mitochondrial Ca^2+^ uptake^86,87^, suggesting that either of these proteins may be involved in MCU-independent Ca^2+^ uptake. Hence, in future studies it will be important to directly evaluate these candidates and others to determine the proteins responsible for AMCU and their role in mitochondria-dependent cell death in in vivo.

## Methods

### Ethics declaration

All experimental procedures with animals were approved by the Institutional Animal Care and Use Committee of Cincinnati Children’s Medical Center, protocols IACUC 2019–0047 and 2021–0047. We have complied with the relevant ethical considerations for animal usage overseen by this committee as described in the animal section of the methods. No human participants or samples were utilized in this study. All animal experimentation followed the recommendations in the ARRIVE guidelines.

### Animal models

To study the role of the MCU-complex we utilized muscle-specific *Mcu* or *Mcub* gene deletion using the previously described *Mcu-loxP* (*Mcu*^*F*^) mice^14,74^ and *Mcub-loxP (Mcub*^*F*^*)* mice^18,37^ and the *Myod*^*Cre*^ knock-in mouse which possess the *Cre* recombinase cDNA inserted into the muscle-specific *Myod* genetic locus^31^ to generate *Mcu*^*F/F*^*-Myod*^*Cre*^ and *Mcub*^*F/F*^*-Myod*^*Cre*^ mice that are homozygous for their respective *loxP* alleles and heterozygous for the *Myod*^*Cre*^ allele. We crossed these lines to a mouse model of DMD that harbors a spontaneous null mutation in the dystrophin gene (Jackson lab strain: *C57BL/10ScSn-Dmd*^*mdx*^*/J*)^30^, to generate dystrophic muscle-specific deletion mice for *Mcu* (*Mcu*^*F/F*^*-Myod*^*Cre*^*-mdx*) and *Mcub* (*Mcub*^*F/F*^*-Myod*^*Cre*^*-mdx*). All experimental mice were subjected to genotyping wherein genomic DNA was isolated from a small tail biopsy and subsequent PCR experiments were done to confirm homozygosity of the relevant *loxP* alleles (*Mcu-loxP* or *Mcub-loxP*), the presence or absence of the *Myod*^*Cre*^ gene insertion, and the presence or absence of the *mdx* mutant allele in males (on the X chromosome). We utilized the following PCR primers: *Mcu-loxP* (5’-GAAGGCCTCCTGTTATGGAT-3’/5’-CCAGCTTGGTGAAGCCTGAT-3’), *Mcub-loxP* (5’- GGCCATGGCAACTTACAAAA-3’/5’-GCCTGTTGGAGGAGAGATGT-3’/5’-GAACTTCGGAATAGGAACTTCG-3’), *Myod*^*Cre*^ (5’-GCGGATCCGAATTCGAAGTTCC-3’/5’-TGGGTCTCCAAAGCGACTCC-3’), *mdx* (5’- GCGCGAAACTCATCAAATATGCGTGTTAGTGT-3’/5’- CGGCCTGTCACTCAGATAGTTGAAGCCATTTTA-3’/5’- GATACGCTGCTTTAATGCCTTTAGTCACTCAGATAGTTGAAGCCATTTTG-3’).

All experimental procedures with animals were approved by the Institutional Animal Care and Use Committee of Cincinnati Children’s Medical Center, protocols IACUC 2019–0047 and 2021–0047. Mice were housed in an AAALAC certified vivarium at 21–22 °C, 40–60% humidity, on a 12-h light/dark cycle, and had free access to food and water ad libitum. Cages were changed every 2 weeks and mice were assessed daily for health and well-being by veterinary technicians at Cincinnati Children’s Hospital Medical Center. Housing conditions and husbandry at Cincinnati Children’s Hospital conformed to AAALAC standards as well as the standard guidelines from the Office of Laboratory Animal Welfare (OLAW, https://olaw.nih.gov/guidance/topic-index/animal-use.htm). All animal experimentation related to this study was approved by the Office of Research Compliance and Regulatory Affairs. Moreover, all animal experimentation followed the recommendations in the ARRIVE guidelines.

The number of mice used in this study reflects the minimum number needed to achieve statistical significance based on experience and previous statistical power analysis. Blinding was performed for some experimental procedures with mice, although blinding was not possible in every instance. Only male mice were used in this study to model the human disease in X-linked DMD that primarily affects males. No male animals were excluded from analysis. Randomization of mouse groups was not performed because mice were genetically identical and littermates were used whenever possible.

### Mitochondrial isolation

Mitochondria were isolated in MS-EGTA buffer [225 mM mannitol, 75 mM sucrose, 5 mM Hepes, and 1 mM EGTA (pH 7.4); Sigma-Aldrich]. Quadriceps or heart tissue was minced into 2 mm by 2 mm pieces in 4 mL of MS-EGTAS supplemented with 0.2 mg/mL Trypsin (Worthington; 3703; 187 u/mgP) and digested for 5 min. Digestion was stopped by the addition of 10 mL of 0.2% BSA MS-EGTA. Seven milliliters of the resulting buffer containing the majority of tissue was then homogenized using a glass and Teflon Potter–Elvehjem tissue homogenizer on ice (8 to 15 strokes, depending on the tissue). Heart and muscle tissue homogenates were then subjected to a 2000 xg centrifugation for 5 min (1x), and the supernatants were then centrifuged at 11,500 xg for 10 min. Liver and brain tissues were resuspended in 7 ml of MS-EGTA buffer, minced into 2 mm by 2 mm pieces, and homogenized using a glass and Teflon Potter–Elvehjem tissue homogenizer on ice (8 to 10 strokes, depending on the tissue). Liver and brain tissue homogenates were then subjected to a 800 xg centrifugation for 5 min (1x), and the supernatants were then centrifuged at 10,000 xg for 10 min. Following centrifugation mitochondria were suspended in KCl buffer [125 mM KCl, 20 mM Hepes, 2 mM MgCl_2_, 2 mM KH_2_PO_4_, and 40 μM EGTA (pH 7.2); Sigma-Aldrich] and either utilized in a functional assay or spun down (5000 xg for 5 min) and snap frozen in liquid nitrogen for further analysis.

### Western blotting

Western blots were performed using isolated mitochondrial lysates, tissue homogenates, or cell lysates as specified in the figure caption. Following mitochondrial isolation, the snap frozen mitochondrial pellets were suspended in Radioimmunoprecipitation assay buffer (RIPA buffer) [10 mM Tris–HCl, 150 mM NaCl, 4% Glycerol, 0.5 mM NaMetabisulfite, 1% Triton X-100, 0.1% NaDeoxycholate, 0.05% SDS (pH 7.5); Sigma-Aldrich] containing protease inhibitor cocktails (Roche; 4693124001). The samples were then sonicated and centrifugated to remove the insoluble fraction. The insoluble fractions were discarded following centrifugation. SDS sample buffer [50 mM Tris–HCl, 2% SDS, 10% glycerol, 1% β-mercaptoethanol, 12.5 mM EDTA, 0.02% bromophenol blue (pH 6.8); Sigma-Aldrich] was added to the lysates, and samples were boiled for 5 min. The samples were then loaded onto 10% acrylamide gels and transferred onto polyvinylidene fluoride transfer membranes (MilliporeSigma). For Western blots of whole quadriceps protein extracts, the tissue was snap frozen in liquid nitrogen and then dry homogenized using a frozen mortar and pestle. The resulting muscle homogenate powder was resuspended in RIPA buffer and then treated as above to generate western samples. Cell protein isolates were generated by scraping off the confluent cell monolayer on ice, generating a cell pellet via brief centrifugation at 4 °C, resuspending the pellet in RIPA buffer, and sonication followed by centrifugated to remove the insoluble fraction. Cell protein lysates were then treated as above to generate western samples. The following commercially available primary antibodies were used: MCU monoclonal antibody (Cell Signaling; 14997; 1:1000), GAPDH monoclonal antibody (Fitzgerald; 0RG109A; 1:5000), and Total OXPHOS rodent WB antibody cocktail (that contained antibodies to electron transport chain (ETC) complexes 1–5; specifically Succinate Dehydrogenase Complex Iron-Sulfur Subunit B (SDHB) from complex II was measured) (Abcam; ab110413; 1:1000). Additionally, we utilized a custom-made MCUb rabbit primary polyclonal antibody (produced by YenZym Antibodies LLC; 1:250)^18^. Fluorescence-based secondary antibodies were used against different species of antibodies and incubated at room temperature for 1 h (LI-COR Biosciences; 926–32211 or 925–68070; 1:10,000). Membranes were imaged using an Odyssey CLx Imaging System (LI-COR Biosciences). Supplemental Fig. S2 shows full gel images of all western blotting used in the manuscript.

### Isolated mitochondrial Ca^2+^ uptake, swelling, and overload assays

All isolated mitochondrial experiments were performed using a dual-detector (one to measure fluorescence and the other to measure absorbance), single-cuvette-based fluorometric system (Horiba Scientific, PTI QM-800). For assays utilizing mitochondria isolated from skeletal muscle, heart, liver, or brain two milligrams of isolated mitochondria were utilized in all experiments. All mouse tissue isolated mitochondria experiments utilized mitochondria from 8-week-old mice. For assays utilizing mitochondria isolated from HEK293T cells one milligram of isolated mitochondria were used. To assess mitochondrial uptake and MPTP sensitivity we utilized the “standard” calcium retention capacity (CRC) assay ^7^. Briefly, isolated quadriceps mitochondria were loaded into the cuvette along with 250 nM cell-impermeant Calcium Green-5N (Invitrogen; C3737), 7 mM pyruvate (Sigma-Aldrich), and 1 mM malate (Sigma-Aldrich) and brought up to 1 mL using KCl buffer (as above). Mitochondria were then pulsed with sequential additions of CaCl_2_ (20 μM or 40 µM as specified in the figure caption) at a rate of one pulse every 2 min until MPTP opening occurred as assessed by mitochondrial Ca^2+^ release and/or swelling or by Calcium Green dye saturation. To assess mitochondrial Ca^2+^ uptake in response to “high Ca^2+^ levels” we devised an assay wherein we loaded isolated mitochondria internally with the cell-permeant, mitochondria matrix localizing Ca^2+^ sensor dye Rhod-2-AM. Isolated mitochondria were resuspended in KCl buffer supplemented with 5 µM Rhod-2-AM dye (Invitrogen; R1244) and incubated for 15 min on ice, pelleted via brief centrifugation, subjected to a second 15 min incubation in KCl buffer on ice to wash out unincorporated dye, pelleted a second time, resuspended in 1 mL KCl supplemented with 7 mM pyruvate and 1 mM malate, and transferred to the assay cuvette. For isolated tissue mitochondria: Rhod-2-AM loaded mitochondria were subjected to a high Ca^2+^ stimulation protocol wherein they were treated with ten pulses of 2 mM CaCl_2_ at 1-min intervals followed by five additional minutes of recording. Mitochondrial swelling experiments were performed either simultaneously with CRC experiments or as separate assays utilizing isolated mitochondria resuspended in 1 mL KCl buffer supplemented with 7 mM pyruvate and 1 mM malate and subjected to 2 mM CaCl_2_ pulses at 1-min intervals until either swelling was observed or ten pulses had been delivered, as specified in the figure caption. In some experiments mitochondria were treated with the inhibitors 5 μM Ru360 (Calbiochem; 557440), 1 μM Ruthenium-Red (Abcam; ab120264), or 10 μM of the chemical Ca^2+^ ionophore ETH-129 (Sigma-Aldrich; 21193) as specified in the figure caption.

Mitochondrial Ca^2+^ load was measured as previously described^5^. Briefly, we measured Ca^2+^ mitochondrial load using the above system and experimental conditions, however mitochondria were suspended in modified KCl buffer lacking EGTA [125 mM KCl, 20 mM Hepes, 2 mM MgCl_2_, 2 mM KH_2_PO_4_ (pH 7.2); Sigma-Aldrich]. To then assess total matrix Ca^2+^, 2 mg of isolated quadriceps mitochondria were treated with a membrane-permeabilizing agent, 40 μM alamethicin (Santa Cruz Biotechnology; CAS 27061-78-5), and the amount of matrix Ca^2+^ released was assessed by the increase in Calcium Green fluorescence.

### Immunohistochemistry and histological analysis

Histological sections (10 µm) were collected from frozen skeletal muscles using a cryostat. For laminin staining to assess myofiber cross-sectional areas, frozen sections were fixed in 4% paraformaldehyde (PFA) for 10 min, washed in 1X phosphate buffer saline (1X PBS), and maintained in blocking buffer for 30 min [10% goat serum and 0.4% triton X diluted in 1X PBS]. Slides were stained overnight at 4 °C with anti-laminin antibody (Sigma; L9393; 1:200) diluted in staining solution [1% bovine serum albumin, 0.04% triton X diluted in 1X PBS]. Slides were then stained Alexa-568 goat anti-rabbit IgG secondary antibody (Invitrogen, A-11036; 1:300) for 1 h at room temperature (RT). Nuclei were visualized by a 15 min 4′,6-diamidino-2-phenylindole (DAPI) stain (Invitrogen; D3571; 1:5000). For Myh3 staining frozen sections were fixed with cold acetone for 10 min, air dried for 30 min, washed with 1X PBS, and blocked with 2% bovine serum albumin (BSA) in 1X PBS for 1 h. Slides were stained overnight at 4 °C with anti-Myh3 antibody (Developmental Studies Hybridoma Bank; F1.652, 1:20) diluted in staining solution. Slides were then stained with Alexa-488 goat anti-mouse IgG1 secondary antibody (Invitrogen; A-21121; 1:400) for 1 h at room temp. Slides were subsequently stained with wheat germ agglutinin (WGA) conjugated to Alex Fluor 488 (Invitrogen; W11261; 1:200) and DAPI for 20 min to visualize myofiber outlines and nuclei, respectively. Immunofluorescence images were captured using a Nikon Eclipse Ti microscope using NIS Elements AR 4.13 software. Regenerating myofibers were assessed by measuring Myh3 positive muscle area from an entire quadricep section at the mid-belly. The minimal Feret’s diameter was determined from laminin-stained whole quadriceps muscle and one thousand fibers were measured per quadricep section. All analyses were performed using NIS Elements AR 4.13 software and conducted in a blinded fashion whereby the experimenter was only aware of the genotypes following quantification.

Histological cross-sections (10 μm) were collected from quadriceps using a cryostat and stained for H&E muscle pathology. The rate of myofiber central nucleation was quantified from one 10X magnification micrograph taken from histological sections of the quadriceps at the mid-belly. One thousand fibers were measured per quadricep section. All analyses were performed in a blinded fashion whereby the experimenter was only aware of the genotypes following quantification.

### Forced treadmill running and isometric force measurement

To assess the exercise capacity of mice, treadmill running was performed as previously described^26^. Mice were run in an electrically driven 6-lane treadmill (Columbus Instruments International; Exer-3/6 Treadmill) at a 5° decline. Mice were given a 10 min training regimen at 6 m/min to familiarize them with the environment and shock grids adjustable from 0–2.0 mA. The speed was then increased in increments of 2 m/min every 3 min to a maximum speed of 18 m/min. Exhaustion was assessed as greater than 10 consecutive seconds on the shock grid without attempting to reengage the treadmill which was considered an experimental endpoint.

To directly measure muscle function, mice were utilized in isometric muscle force generation experiments. Mice were anesthetized by intraperitoneal injection of pentobarbital (6 mg/100 g body weight). The distal tendon of the tibialis anterior (TA) muscle was exposed, and 4–0 nylon suture was tied at the muscle–tendon interface. The knee and foot of the mouse were secured to a platform and the tendon was mounted to a servomotor (Aurora Scientific; Model 305C). The TA muscle was stimulated using two intramuscular electrodes placed on either side of the peroneal nerve at range of 25–200 Hz with a bi-phase high-power stimulator (Aurora Scientific; Model 701C). Stimulation voltages and optimal muscle length were adjusted to produce maximum isometric twitch force. A series of six consecutive tetanic isometric contractions were performed with a 2 min rest period between each contraction. Specific force was measured by dividing the active force by the muscle's physiologic cross-sectional area and the percent drop in force was determined by taking the difference in force between contractions 1 and 6. The experimenter was blinded to the genotypes during the collection of these data.

### Serum creatine kinase measurement

Blood samples from mice were collected from the abdominal aorta of anesthetized mice and plasma was isolated by centrifugation at 5000 xg for 15 min at 4 °C. Serum creatine kinase measurement was performed on a Roche Cobas c311 clinical chemistry analyzer (Roche) by a trained observer blinded to genotype.

### Mitochondrial Ca^2+^ uptake measurement in intact cells

To measure MCU-independent Ca^2+^ uptake in cells we generated a HEK293T *MCU* gene deleted cell line (human origin) utilizing a CRISPR-Cas9 strategy followed by clonal selection and screening for DNA recombination, loss of MCU protein, and loss of acute Ca^2+^ uptake in a standard CRC assay. We utilized Lipofectamine 3000 transfection reagent (ThermoFisher; L3000001) to deliver a mammalian expression plasmid containing the mitochondria targeted Ca^2+^ sensor protein mitoR-GECO^88^ under the control of a CMV promoter (Addgene; #46,021) into wildtype or *MCU*^*-/-*^ HEK293T cells. Twenty-four hours after transfection cells were seeded into glass bottom culture dishes for imaging (MatTek). Forty-eight hours after transfection mitoR-GECO transfected cells were imaged for mitoR-GECO fluorescence (Excitation: 550 nm; Emission: 600 nm) via confocal microscopy (Nikon A1R inverted LUNV confocal laser scanning microscope). Live cells were imaged continuously for 5 min and treated with 250 µM ATP (Invitrogen) to elicit a cellular Ca^2+^ response following a 30 s baseline period. Data were then analyzed for mitoR-GECO intensity throughout the imaging period for each mitoR-GECO positive cell in the imaging field (~10–30 cells per field) utilizing NIS Elements AR 4.13 software. To eliminate cytosolic off-target fluorescent signal we utilized the optical properties of confocal microscopy to restrict the z-axis and utilized the data analysis software to restrict data in x/y-axis to only include mitochondrial regions (as defined by mitoR-GECO positive cellular regions at baseline). We also excluded rare cells from our analysis with obvious off-target cytosolic Ca^2+^ signals. In some experiments cells were pre-treated with 25 μM ruthenium red (RuRed) for 15 min and maintained on the drug during the experiment.

### Statistics

All results are presented as mean ± SEM unless otherwise specified. Data from each experiment was subjected to a Brown-Forsyth test for unequal variance prior to additional statistical analysis. For datasets with equal variance statistical analysis was performed using one-way ANOVA with post hoc Dunnett’s test for multiple comparisons of 3 or more independent groups. Two-way ANOVA with post hoc Dunnett’s or Tukey’s tests for multiple comparisons was utilized where appropriate. For datasets with unequal variance a nonparametric Kruskal–Wallis test was utilized to assess statistical significance. All statistics were performed using GraphPad Prism 9 and values were considered statistically significant when p ≤ 0.05. No statistical analysis was used to predetermine sample size although previous power analysis of similar experimental variables in mice did direct selection of group sizes. The investigators were not blinded to allocation during all experiments but were blinded in specific experimental outputs as noted.

### Supplementary Information

Supplementary Information.

## Data Availability

All data are provided within the manuscript or supplementary information files for data directly related to this study.
