# Supplementary material for: MCU-independent Ca2+ uptake mediates mitochondrial Ca2+ overload and necrotic cell death in a mouse model of Duchenne muscular dystrophy
Source: Sci Rep. 2024 Mar 21;14:6751. doi: 10.1038/s41598-024-57340-3 (PMC10957967; doi:10.1038/s41598-024-57340-3)

Supplementary Materials

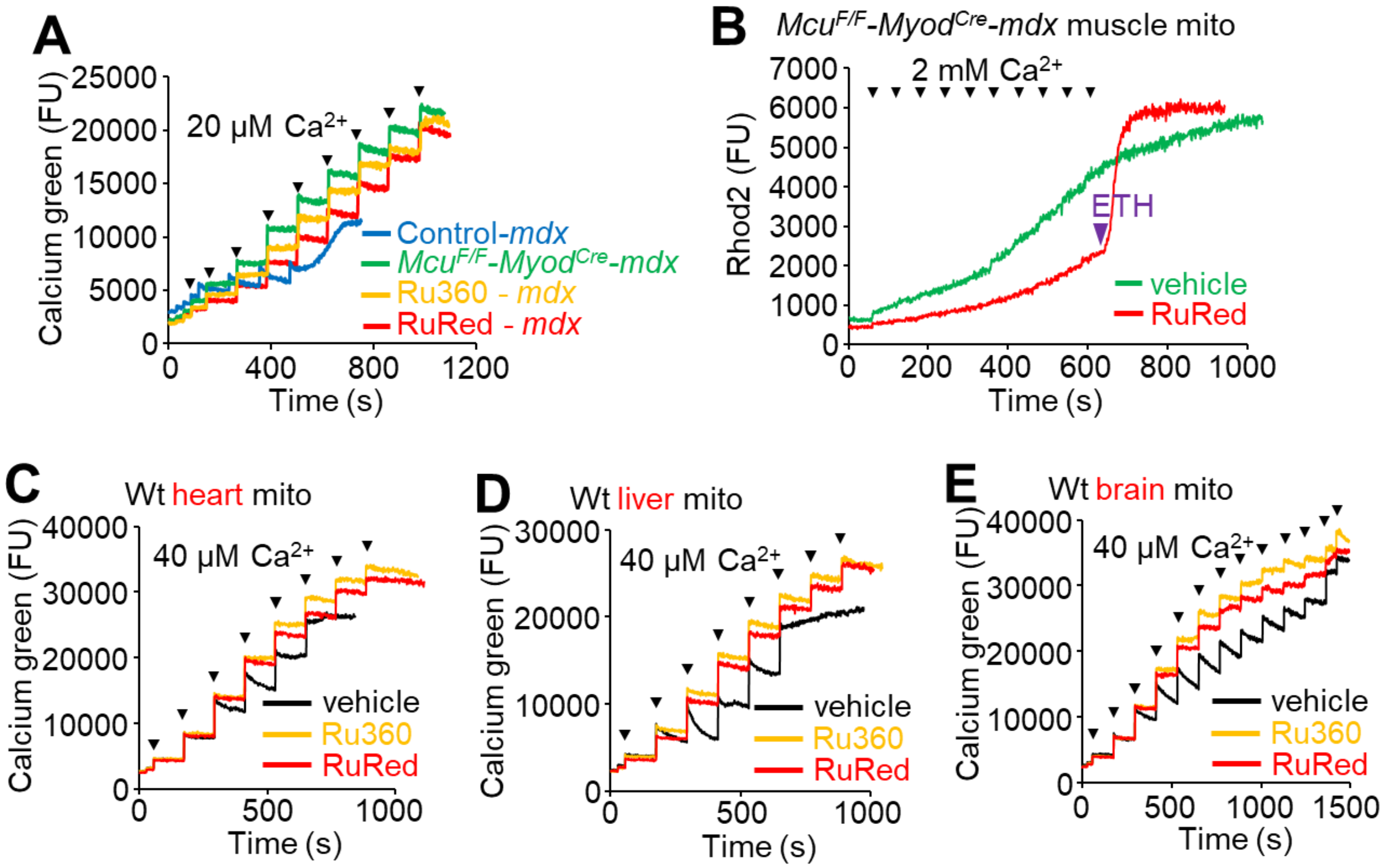

Supplemental Figure S1: Effects of Ru360 and ruthenium red (RuRed) treatment on Ca^{2+} uptake in isolated mitochondria. (A) Representative mitochondrial CRC assay of isolated quadriceps mitochondria from *mdx,* and *Mcu$^{F/F}$-Myod^{Cre}-mdx* mice. Two milligrams of mitochondria were pulsed 10x with 20 µM additions of $CaCl_2$ (arrows). *mdx* mitochondria were treated with 5 µM Ru360 or 1 µM RuRed as indicated. Data are plotted as calcium green fluorescence units (FU). The presented data are representative of at least 3 independent experiments utilizing mitochondria from 3 separate mitochondrial isolations from different mice of the indicated genotypes. This is true for all representative data presented throughout this figure. **(B)** Representative mitochondrial Ca^{2+} uptake in isolated *Mcu$^{F/F}$-Myod^{Cre}-mdx* quadriceps mitochondria treated with 10 pulses of 2 mM $CaCl_2$ (arrows). *Mcu$^{F/F}$-Myod^{Cre}-mdx* mitochondria were treated with 1 µM RuRed followed by 10 µM ETH-129. Purple arrow indicates time of ETH-129 addition. Data are plotted as Rhod2 fluorescence units (FU). **(C)** Representative mitochondrial CRC assay of isolated heart mitochondria from wildtype mice treated with 5 µM Ru360 or 1 µM RuRed as indicated by the figure legend. Two milligrams of mitochondria were treated with 10 pulses of 40 µM $CaCl_2$ (arrows). Data are plotted as calcium green fluorescence units (FU). **(D)** Representative mitochondrial CRC assay of isolated liver mitochondria from wildtype mice treated with 5 µM Ru360 or 1 µM RuRed as indicated by the figure legend. Two milligrams of mitochondria were treated with 10 pulses of 40 µM $CaCl_2$ (arrows). Data are plotted as calcium green fluorescence units (FU). **(E)** Representative mitochondrial CRC assay of isolated brain mitochondria from wildtype mice treated with 5 µM Ru360 or 1 µM RuRed as indicated by the figure legend. Two milligrams of mitochondria were treated with 10 pulses of 40 µM $CaCl_2$ (arrows). Data are plotted as calcium green fluorescence units (FU).

Supplemental Figure S2: Uncropped Blots

Figure 1A

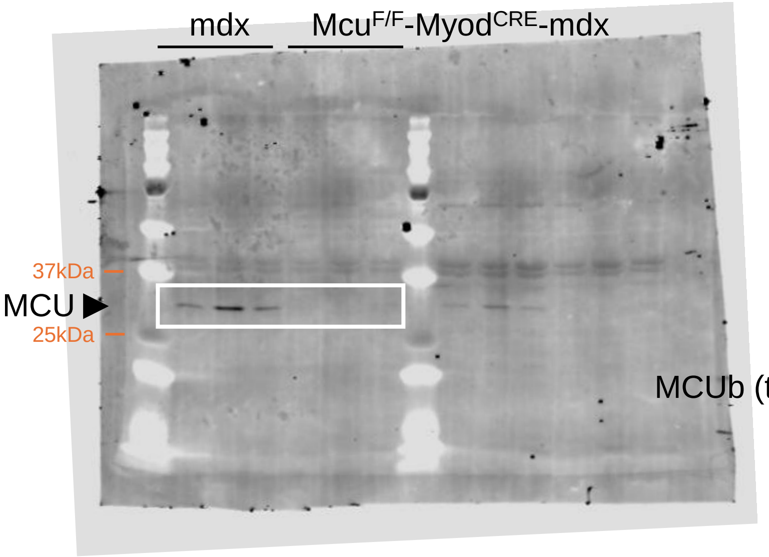

Figure 3B

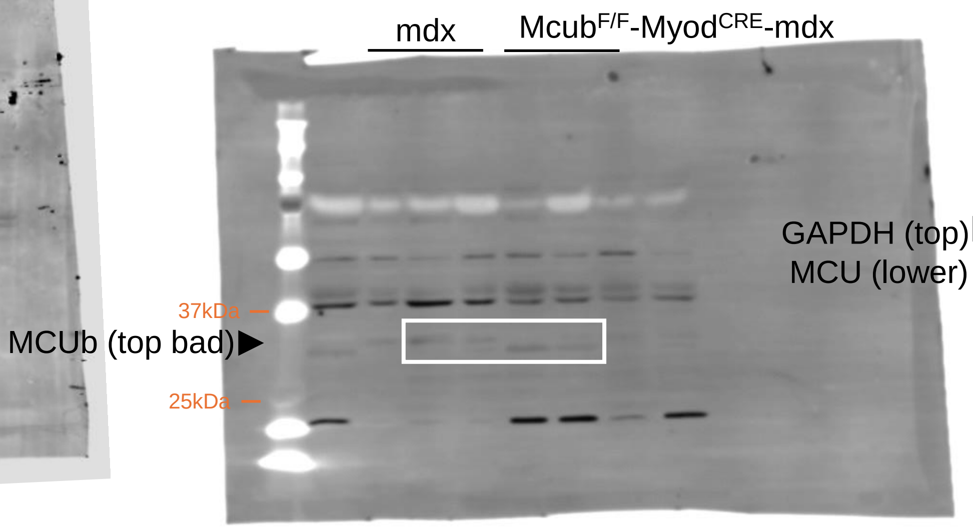

Figure 6A

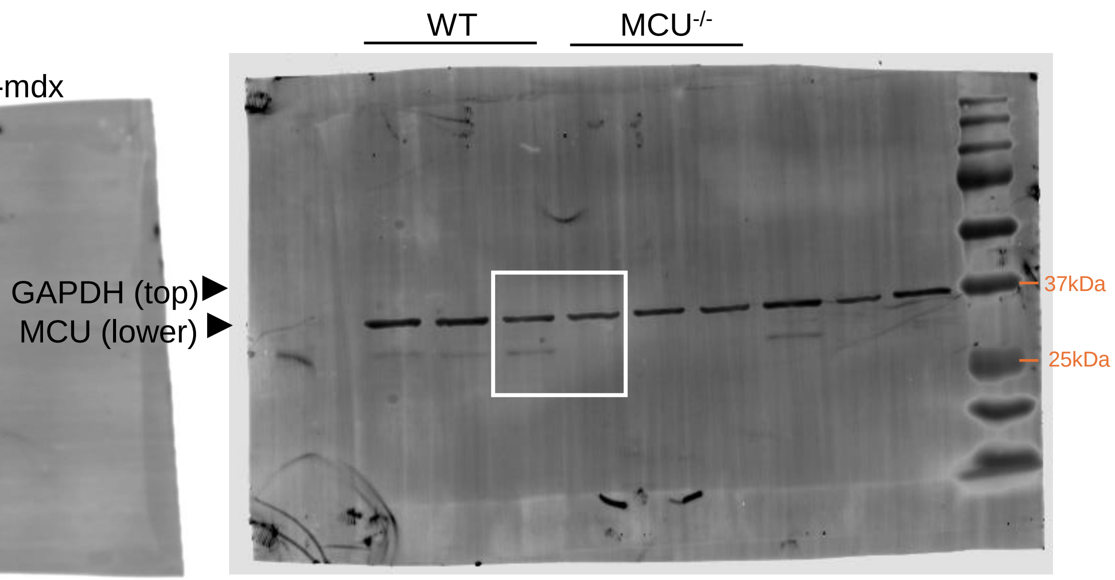

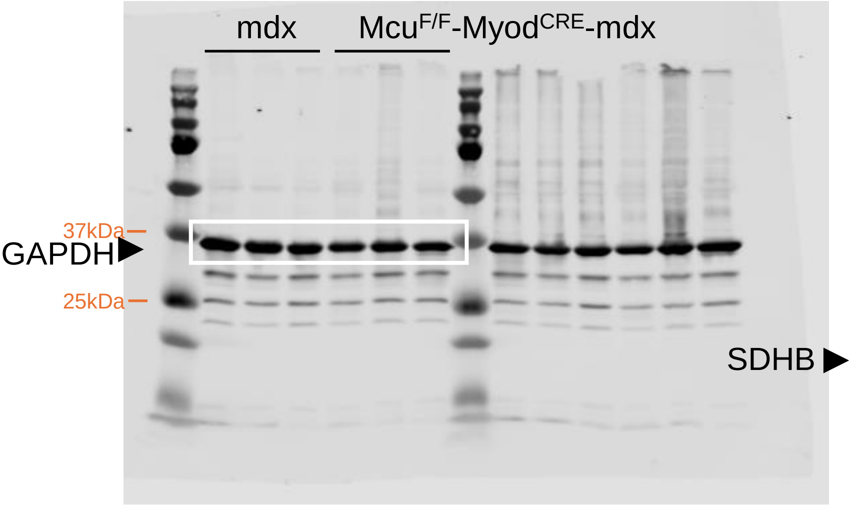

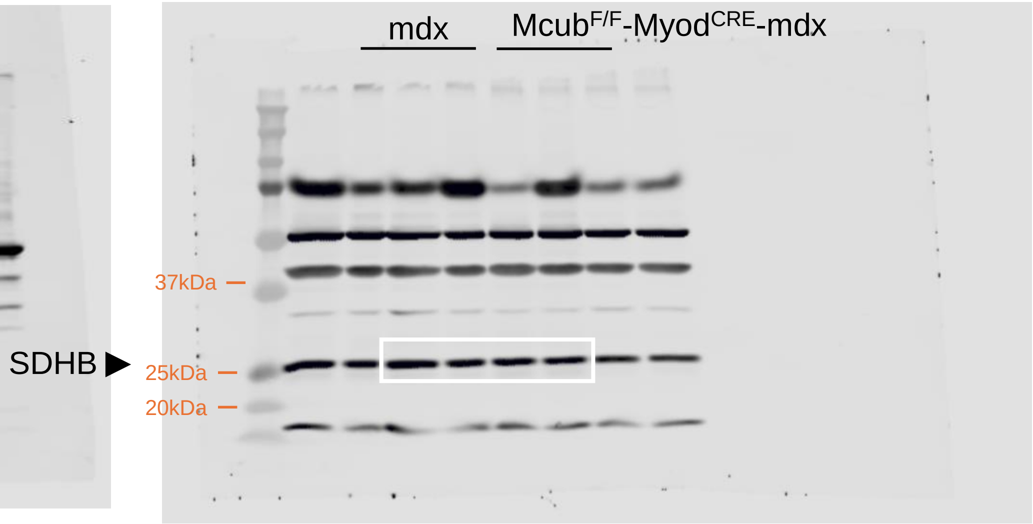

Supplement: Supplementary file 1 — Supplementary Information. [file 41598_2024_57340_MOESM1_ESM.pdf]
